# Renin-Angiotensin System Responds to Prolonged Hypotensive Effect Induced by Mandibular Extension in Spontaneously Hypertensive Rats

**DOI:** 10.3389/fphys.2018.01613

**Published:** 2018-11-15

**Authors:** Laura Sabatino, Chiara Costagli, Dominga Lapi, Cristina Del Seppia, Giuseppe Federighi, Silvana Balzan, Antonio Colantuoni, Giorgio Iervasi, Rossana Scuri

**Affiliations:** ^1^Institute of Clinical Physiology, National Research Council, Pisa, Italy; ^2^Department of Translational Research on New Technologies in Medicine and Surgery, University of Pisa, Pisa, Italy; ^3^Department of Clinical Medicine and Surgery, “Federico II” University Medical School, Naples, Italy

**Keywords:** renin-angiotensin system, AT1R, AT2R, MAS1, mandibular extension

## Abstract

There is an ongoing interest in the renin-angiotensin system (RAS) contribution either to pathological mechanisms leading to hypertension (mainly regarding the ACE/AngII/AT1R axis), or, to RAS protective and pro-regenerative actions, primarily ascribed to the mediation of the AT2R and the MAS1 receptor. In the present study, we evaluated the modulation of gene expression and protein levels of “deleterious” (ACE/AngII/AT1R) and “protective” [ACE/AngII/AT2R and ACE2/Ang(1-7)/MAS1 arms] RAS components in parietal and frontal areas of cerebral cortex of spontaneously hypertensive rats (SHRs), after two periods of mandibular extensions (MEs). Blood pressure, BP and heart rate, HR were also measured. While no significant changes in BP and HR were present in the sham operated (SO) group, in rats after two MEs (2-ME rats), BP displayed a marked decrease (*p* < 0.001) at ME2, and remained then stably low for the subsequent observation period. In gene expression analysis, in SHRs undergoing two MEs, either in parietal or frontal cortex, we did not observe any significant variation of AT2R and ACE2 with respect to SO rats. In contrast, we observed a decrease in Mas1 gene expression in parietal area (*p* < 0.01) and an increase in frontal region (*p* < 0.01). AT1R and ACE gene expression was significantly higher in 2-ME rats than SO in parietal cortex (*p* < 0.05) but no difference was observed in the frontal area. Concerning protein levels, in parietal area, AT1R and AT2R did not change whereas MAS1 significantly decreased in 2-ME rats (*p* < 0.05). In frontal area, both AT1R and AT2R significantly decreased in 2-ME rats (*p* < 0.05), whereas MAS1 did not significantly change. Gene expression analysis in normotensive (NT) rats revealed the non-detectability of AT1R in both parietal and frontal zone. In parietal area, AT2R (*p* < 0.0001) and Mas1 (*p* < 0.01) were significantly decreased in 2-ME NT rats, when compared to SO, and ACE and ACE2 resulted not detectable whereas there was some expression of these genes after 2-ME procedure. In conclusion, our data in rat models indicated that a 2-ME procedure induced a hypotensive response and that a modulation of gene expression and protein levels of RAS components occurred in different cerebral cortex areas.

## Introduction

The facial area is an important site of autonomic reflexes involving parasympathetic and sympathetic nervous systems in the human and these reflexes have trigeminal fibers as afferent branches. Therefore, stimulation of specific facial regions may induce reflex with certain important implications (Brunelli et al., 2012). Recently, we showed that, in normotensive anesthetized rats and in normotensive humans, a mandibular extension (ME) induced a reduction in blood pressure (BP) (Lapi et al., 2013, 2014; Del Seppia et al., 2016, 2017). Moreover, in normotensive rats, this effect was abolished by bilateral peripheral trigeminal section, thus indicating a fundamental role of trigeminal nerve in the hypotensive ME-induced response (Lapi et al., 2013). Furthermore, we have also shown that two periods of MEs in the normotensive rat prolongs the effects of a single ME on BP, heart rate (HR) and vasodilation, modulating the pial arteriolar tone by the increase of endothelial activity (Lapi et al., 2014). Therefore, ME might represent a procedure useful in the control of the systemic BP and, consequently, of the organ perfusion, in particular of the brain and vasculature in cardiovascular diseases, such as hypertension.

Since renin-angiotensin system (RAS) is known to play an important role in the pathophysiology of hypertension, in the present study, we evaluated the gene and protein modulation of RAS after two periods of MEs in a spontaneous hypertensive rat (SHR) model. Furthermore, we evaluated gene expression of RAS components in a group of normotensive (NT) rats undergoing ME procedure, in order to verify that observations on SHRs were effectively related to the hypertensive condition. The molecular analysis was carried out on brain parietal area, where principal trigeminal afferent fibers project, and on brain frontal area, less directly involved in trigeminal nerve functions. It is commonly believed that the effects of AngII (Angiotensin II) on brain depend mostly on AT1R receptor stimulation and that excessive brain AT1R activity correlates with hypertension, heart failure, brain ischemia and more in general, with neurodegenerative disorders (Jackson et al., 2018). However, in the last decades, protective action of the RAS in the nervous system have been investigated and AT2R and MAS1 receptor have been demonstrated to counteract, in most cases, AT1R inflammatory, fibrotic and neurogenerative effects (Horiuchi and Mogi, 2015, Prestes et al., 2017). MAS1 receptor is the main mediator of Ang(1-7) peptide, synthesized by angiotensin-converting-enzyme 2 (ACE2) through the hydrolysis of AngII (Villela et al., 2015). Ang(1-7) has been proven to have important vasodilator, diuretic and natriuretic effects, inhibiting cell growth and norepinephrine release and increasing bradykinin effect (Santos et al., 2003). Aim of the present study is to focus on the efficacy of a repeated ME protocol on main physiological parameters (BP and HR) and the possible effects on RAS deleterious (ACE/AngII/AT1R) and protective (ACE2/Ang(1-7)/MAS1 or ACE/AngII/AT2R) arms in two different rat cortical regions.

## Materials and Methods

### Animals and Housing

We used five 3–4 months old male spontaneously hypertensive rats (SHR, 250–300 g) and six 3–4 months old male normotensive rats (NT, 300 g) from Charles River (Calco, SO, Italy) that were housed in polyethylene cages, under a 12/12 h light/dark cycle (light 8:00–20:00 h) at constant temperature (24 ±1°C) and humidity (60 ± 5%) with free access to food and water.

Experimentation was carried out in accordance with the recommendations in the Guide for the Care and Use of Laboratory Animals of the National Institute of Health. The protocol was approved by the Committee on the Ethics of Animal Experiments of the University of Pisa and Ministry of Health (Permit Number: 157/2017-PR). All surgery was performed under alpha-chloralose and urethane anesthesia and all efforts were made to minimize suffering.

### Surgical Procedure

All rats were anesthetized for the whole duration of the experiment. Anesthesia, tracheotomia, intubation and mechanical ventilation was performed as previously described (Lapi et al., 2014). End-tidal CO_2_ was continuously measured by a CO_2_ analyzer and a respirator (RAT-MOUSE DIGITAL VENTILATOR R-415 2-Biological Instrument, Florence, Italy) was adjusted to maintain end-tidal CO_2_ between 4.5 and 5.0% and to keep arterial blood gas tension within the normal range.

A polyethylene cannula was placed in the left femoral artery to measure MABP.

The rats were secured to a heating stereotaxic frame to monitor rectal temperature and maintain the body temperature at 37.0 ± 0.5°C measured with a rectal probe.

At the end of the experiment, all rats were sacrificed by decapitation and samples of frontal and parietal cortex were rapidly removed and stored at -80°C until the use.

### Mandibular Extension

**FIGURE 1:**
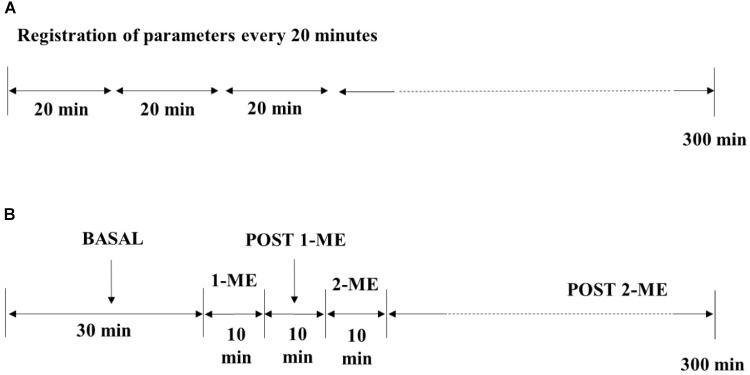
Experimental procedure on SO **(A)** and 2-ME **(B)** SHRs.

To induce ME, was used a home-made U-shaped dilatator appropriately designed for rats and described previously (Lapi et al., 2013, 2017). All ME lasted for 10 min. Both SHRs and NT rats were divided in two groups: 2-ME and sham-operated (SO) control rats. 2-ME rats were subjected, after a 30 min of basal parameters acquisition, to a first ME of 10 min (ME1) followed by a rest of 10 min and a second ME of 10 min (ME2). Rats were then observed for further 240 min before they were sacrificed. BP and HR were constantly measured. Control SHRs underwent surgery without ME procedures (SO), and BP and HR parameters were observed as long as 300 min, so to complete the recording at the same time as 2-ME rats. The experimental procedure on SO and 2-ME rats are shown in Figures 1A,B, respectively.

### Blood Pressure and Heart Rate Measurements

Mean artery BP was monitored with a transducer, model BLPR2 (World Precision Instruments, Sarasota, FL, United States) connected to an ad-hoc bridge amplifier (home-made) and recorded using LabView software (National Instruments S.R.L., Milan, Italy). The instrument measured BP every 60 s and automatically provided an averaged value every 5 min. ECG recording (at a sampling rate of 1 KHz) was performed with a home-made differential amplifier for biomedical signals and data acquisition was obtained by LabView software.

The signal of HR was analyzed beat-to-beat to quantify changes and BP values recorded every 5 min were stored in a computer for off-line analyses. For statistical analysis and for graphical representations, we used values at intervals of 10 and 20 min. We considered as baseline value, the average of three recordings obtained at 10 min intervals immediately prior to ME1, then we considered recordings at 10 min interval for 30 min (corresponding to ME1, POST ME1 and ME2 periods) and at 20 min interval for the subsequent observation period.

### RNA Extraction and Reverse Transcription

Total RNA was extracted from homogenized brain tissue (parietal and frontal cortex) with a miRNeasy Mini Kit (Qiagen, Milan, Italy), according to the manufacturer’s instructions. The integrity of total RNA was detected by electrophoresis of samples on Gel-Star (Lonza, Germany) stained 1.5% Agarose (Bio-Rad, Milan, Italy) gel and total RNA purity and concentration were evaluated spectrophotometrically (NanoDrop, Celbio, Milan, Italy). The RNA samples were stored at -80°C. A quantity of 1 μg of total RNA obtained from each sample was reverse transcribed with iScript cDNA Synthesis Kit (Bio-Rad, Milan, Italy), according to the manufacturer’s instructions.

### Quantitative Real-Time PCR Analysis of Gene Expression

Real-Time PCR reactions were carried out in a 384-well CFX384 RT-PCR System (Bio-Rad, Milan, Italy). As previously described (Sabatino et al., 2015), each reaction was performed 10 μl reaction mixture, including 20 ng of template cDNA, 0.5 μM of each primer and 2X iTaq Universal Sybr Green Supermix (Bio-Rad, Milan, Italy). Gene amplification protocol started with 95°C for 30 s, followed by 39 cycles at 95°C for 5 s and 60°C for 15 s. Each assay was performed in triplicates, with negative control. The combination of GeNorm and qBase software technology, following recent guidelines, was used to assess the expression stability of each candidate housekeeping gene and to determine among them (minimum 3 housekeeping genes) the most reliable for sample normalization (Vandesompele et al., 2002). The average Ct values obtained from each triplicate was converted to a relative quantity and analyzed with CFX384 Manager algorithm. Gene stability is expressed by the M value, which is calculated as the average variation between one of the housekeeping genes and all the others analyzed. The most stable housekeeping genes are identified with M value < 0.5. Our analysis indicated that the three most stably expressed housekeeping genes were HPRT-1: hypoxanthine phosphoribosyltransferase I; Hmbs: Hydroxymethylbilane synthase; Papbn-1: Polyadenylate-binding protein 1. Primers details are shown in Table 1. A standard curve for each target and housekeeping gene was evaluated to assess amplification efficiency and linearity.

**Table 1:** Reference and target genes: primer details.

GENE	PRIMER SEQUENCE (5′→ 3′)	LENGHT bp	GenBank n.
Hprt-1	F: CCCAGCGTCGTGATTAGTGATG	110	NM_012583
	R: TTCAGTCCTGTCCATAATCAGTCC		
Hmbs	F: TCTAGATGGCTCAGATAGCATGCA	76	NM_013168
	R: TGGACCATCTTCTTGCTGAACA		
Papbn-1	F: TATGGTGCGACAGCAGAAGA	110	116697
	R: TATGCAAACCCTTTGGGATG		
AT1R	F: TCTGGATAAATCACACAACCCTC	77	NM_030985.4
	R: GAGTTGGTCTCAGACACTATTCG		
AT2R	F: CTGGCAAGCATCTTATGTAGTTC	115	U22663.1
	R: ACAAGCATTCACACCTAAGTATTC		
Mas1	F: ACTGTCGGGCGGTCATCATC	272	NM_012757.2
	R: GGTGGAGAAAAGCAAGGAGA		
ACE	F: TCCTATTCCCGCTCATCT	127	NM_012544.1
	R: CCAGCCCTTCTGTACCATT		
ACE2	F: GAATGCGACCATCAAGCG	228	AY881244
	R: CAAGCCCAGAGCCTACGA		


### Western Blotting

Cell lysate proteins (60 μg) were resolved by Bolt 8% gradient mini gels using the Bolt mini gel electrophoresis system (Life Technologies, Monza, Italy). Gels were blotted onto 0.2 mm PVC-membrane by iBlot Dry Blotting System (Life Technologies, Monza, Italy). Membranes were incubated with specific polyclonal Antibody for AT1R, AT2R, MAS1, GAPDH or HPRT (Abcam, Milan, Italy) and the appropriate secondary G-Immunoglobulin conjugated to horse radish peroxidase (IgG-HRP) was applied. Proteins were visualized with a chemiluminescence assay (Clarity Western ECL Substrate, Bio-Rad, Milan, Italy) and images were acquired and analyzed by Alliance Uvitec Cambridge System. The results were expressed as target protein OD normalized to the reference protein OD. Final data were obtained from an average of three assays.

### Statistical Analysis

All values are expressed as mean ± SEM. For the gene expression data and proteins levels analysis the non parametric Mann-Whitney *U*-test was run using StatView 5.0.1 software (SAS Institute Inc., United States). For BP and HR statistic, the one-way ANOVA for repeated measures and the Holm Sidak *post hoc* test were run using the Sigma Stat package, 3.5 version (Jandel Corporation San Mateo, CA, United States). *p* < 0.05 was considered statistically significant.

## Results

### Mean Arterial Blood Pressure (BP) and Heart Rate (HR)

**FIGURE 2:**
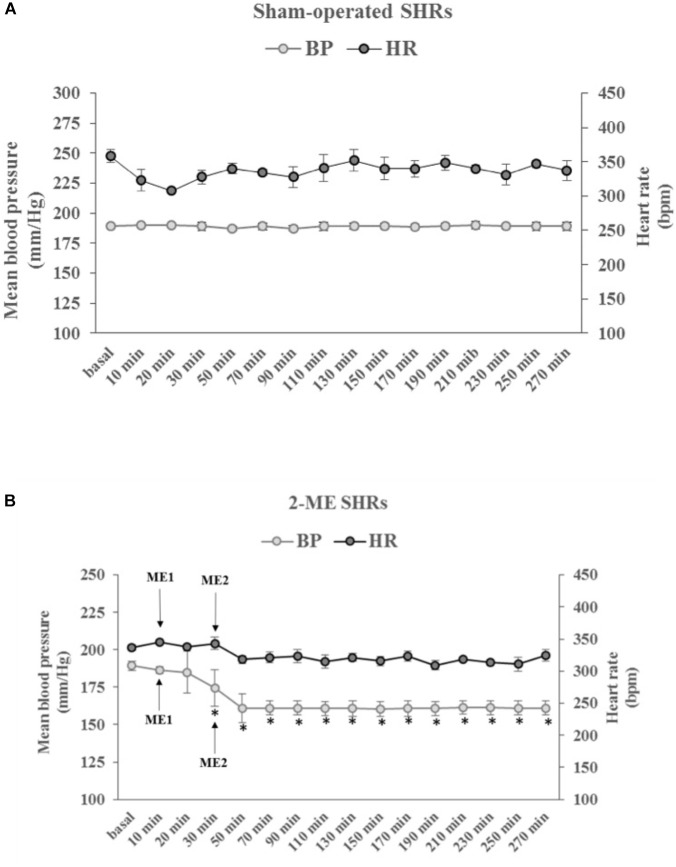
The time-courses, in SHRs, of the mean blood pressure (BP, left ordinate axis) and heart rate (HR, right ordinate axis) in **(A)** SO rats (*N* = 2) and **(B)** 2-ME (*N* = 3) rats. ME1 and ME2 with arrows indicate the timing of ME treatments. Values (means ± SEM) are plotted every 10 min for the first 30 min after baseline values (basal) and every 20 min, thereafter. ^∗^*p* < 0.001 vs. baseline value.

For BP, no significant change was present in the SO SHRs for the entire observation period of 300 min (Figure 2A). In 2-ME rats, BP displayed a significant (*p* < 0.001) and marked decrease after ME2 (from 189 ± 1.45 mmHg -baseline value- to 161 ± 3.22 mmHg). Thereafter, BP remained stably low for the subsequent observation period of 270 min (Figure 2B). For HR, no significant difference was measured in the observation period, either for SO or 2-ME rats (Figures 2A,B).

### Gene Expression in SHRs

**FIGURE 3:**
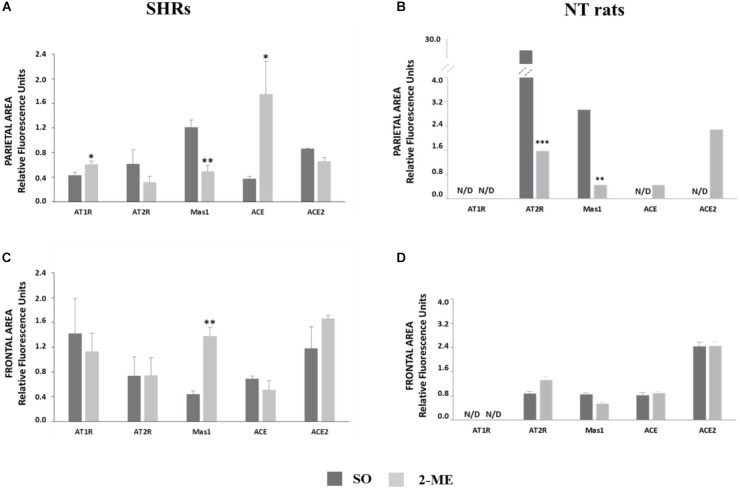
Representation of gene expression of AT1R, AT2R Mas1, ACE and ACE2 in SHRs (*N* = 5) or NT rats (*N* = 6) undergoing a double ME and their comparison with SO in parietal (**A**, SHRs and **B**, NT rats) and frontal (**C**, SHRs and **D**, NT rats) areas. Data are expressed as mean ± SEM. ^∗^*p* < 0,05; ^∗∗^*p* < 0.01; ^∗∗∗^*p* < 0,0001 vs. SO.

In 2-ME SHRs, both in parietal and frontal regions of cerebral cortex, we did not observe any significant variation of AT2R and ACE2 with respect to the SO SHRs. Differently, Mas1 significantly decreased in parietal region (*p* < 0.01) and increased in frontal area (*p* < 0.01), with respect to SO SHR values. AT1R and ACE were significantly higher in 2-ME than in SO rats in parietal brain (*p* < 0.05) but no difference was observed in the frontal area (Figure 3A, parietal area; Figure 3C, frontal area).

### Gene Expression in NT Rats

In SO and 2-ME rats, both in parietal and frontal regions of cerebral cortex, AT1R resulted not detectable (N/D). In the parietal area, AT2R and Mas1 significantly decreased after 2-ME (*p* < 0.0001 and *p* < 0.01, respectively, with respect to SO rats. Moreover, also ACE and ACE2 gene expression was N/D in SO rats whereas some expression levels were observed in 2-ME rats. In the frontal area, in 2-ME rats we did not observe any significant variation of any studied gene with respect to the SO rats. (Figure 3B, parietal area; Figure 3D, frontal area).

### Protein Levels in SHR Rats

**FIGURE 4:**
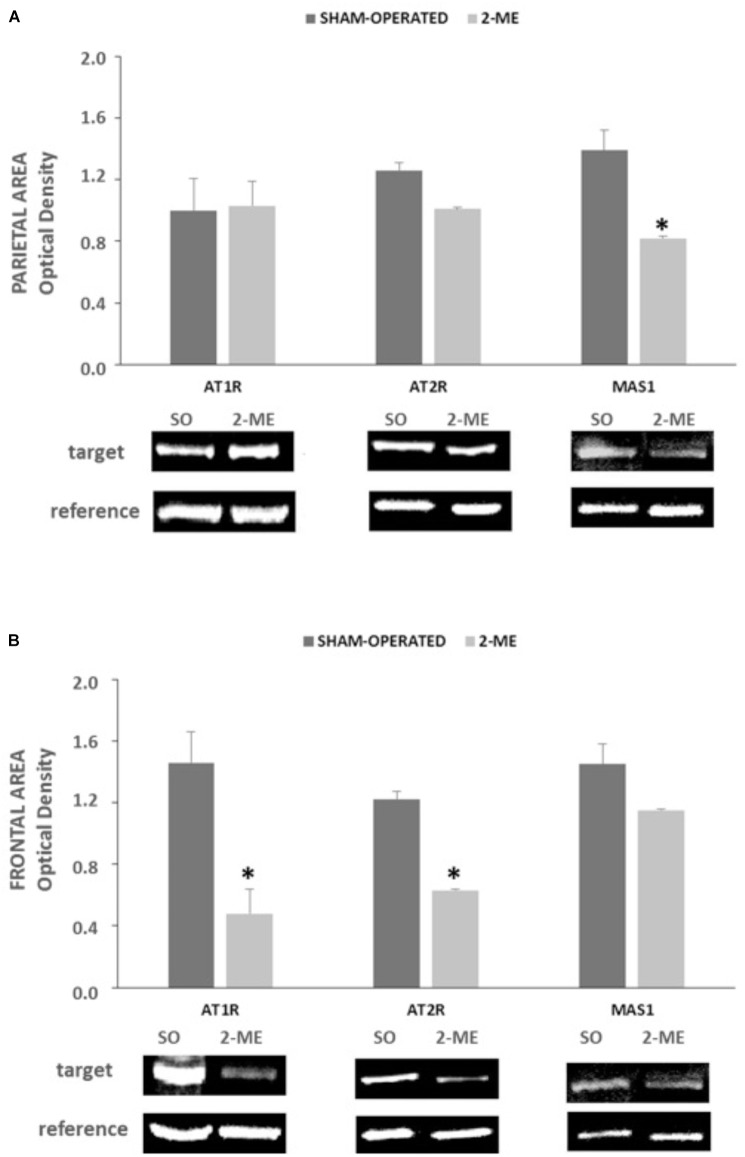
AT1R, AT2R e MAS1 protein levels in SO (*N* = 2) and 2-ME (*N* = 3) rats. Data are represented as mean ± SEM. Histograms representing the Optical Density of target proteins (AT1R, AT2R, and MAS1) normalized to the Optical Density of the housekeeping proteins (GAPDH for AT1R and HPRT for AT2R and MAS1) are represented in panel **A** (data from the parietal area) and panel **B** (data from the frontal area) of SHR cerebral cortex. ^∗^*p* < 0.05 vs. SO.”

In parietal area, protein levels of AT1R and AT2R did not change in 2-ME rats with respect to SO, whereas MAS1 were significantly reduced (*p* < 0.05) (Figure 4A).

In frontal area, both AT1R and AT2R significantly decreased in 2-ME rats with respect to SO rats whereas MAS1 did not significantly change (Figure 4B).

## Discussion

In the present study we evaluated the effects of a repeated ME procedure on modulation of main physiological parameters (BP and HR) and of principal components of RAS in SHRs, focusing primarily on the response in cerebral cortex. Previous studies in normotensive rats, investigating arteriolar tone modifications, have shown that the stimulation of trigeminal nerve by ME procedure determines a prolonged hypotensive effect accompanied by initial rapid vasoconstriction, followed by a prolonged vasodilation y in the pial microcirculation (Lapi et al., 2017). Furthermore, our studies on humans demonstrated that the submaximal mouth opening for a relatively brief time (10 min) was followed by a prolonged hypotensive and bradycardic effect on normotensive volunteers (Del Seppia et al., 2016, 2017). Due to the important role of RAS in the pathophysiology of hypertension, we focused on the gene expression and protein levels of RAS components during ME protocol at cortex level in SHRs and in NT rats.

It is commonly believed that AngII acts via its receptor AT1R to increase BP, stimulating sympathetic effects and vasopressin secretion (Jancovski et al., 2013). However, besides the deleterious involvement of ACE/AngII/AT1R system in BP alteration, it is conceivable that other components of RAS, collectively known as “protective RAS” may be engaged to counteract the hypertensive drive. As far as we know, protective RAS consists of two major arms: ACE/AngII/AT2R and ACE2/Ang(1-7)/MAS1 (Steckelings and Unger, 2012). In the brain, there is some evidence that AT2R and MAS1 not only functionally oppose to AT1R in BP control but also have unique effects that lie outside of AT1R signaling (Horiuchi and Mogi, 2015; Prestes et al., 2017) and that mostly regard cognitive functions (Sakata et al., 2009), cell survival, antioxidant and anti-inflammatory properties (de Kloet et al., 2017). Moreover, until recently, the potential central nervous system-mediated effects of Ang II/AT2R and Ang(1-7)/MAS1 in BP regulation and hypertension have been understudied, and still not well understood (de Kloet et al., 2017). More recently, a better understanding of the distribution of AT2R in the brain was supported by fluorescence *in situ* hybridization studies, which provided a discrete view of the cellular localization of AT2R in this organ (de Kloet et al., 2016). From information available on AT1R and AT2R expression, data in mouse indicated an overlap between the two AngII receptors (Lenkei et al., 1997) and it has been hypothesized that direct antagonistic effects of the two receptors in the same cells use different mechanisms to interfere in the same pathways (Matsuura et al., 2005). In hypertensive rats, the supplemental action of MAS1 receptor and involvement of Ang(1-7) is believed to be crucial in the compensatory activity aiming to restore a normal BP (Lucas et al., 2015). Conversely, in primary murine astrocytes cultures, has been observed that the presence of AT2R is required for Ang(1-7) activity and, reversely, MAS1 is required for beneficial effects of AT2R, suggesting a synergistic interplay between receptors and ligands (Leonhardt et al., 2017). It is known from the literature that SHR hypertensive phenotype in the brain is greatly sustained by an alteration of a balance in ACE/ACE2, and that AngII synthesis is favored with respect to Ang(1-7) generation (Gowrisankar and Clark, 2016). Specifically, in our SHR model, two hypotensive ME procedures were performed, as previously described in normotensive rats (Lapi et al., 2017). These results are in accordance with those obtained in normotensive rats in which a repeated stimulation induced a decline in BP by nearly 20 mmHg (Lapi et al., 2017), and suggest that the hypotensive effect is even greater (about 28 mmHg, Figure 2B) and more prolonged in hypertensive animals. At molecular level, AT1R and ACE gene expression were significantly reduced only in parietal area while they did not change in frontal region. Differently, AT2R and ACE2 gene expression did not vary both in parietal and frontal regions (Figures 3A,C). Furthermore, in the two cortical areas analyzed, an opposite response was observed for Mas1 receptor gene expression, since it was significantly reduced after the hypotensive 2-ME procedure in the parietal region (Figure 3A) and greatly increased in the frontal area (Figure 3C). Since both AT1R and AT2R protein levels significantly decreased in frontal area (Figure 4B), a possible post-translational mechanism, inducing the inhibition of AngII receptors can be hypothesized. Moreover, at protein level, MAS1 was significantly reduced in the parietal area (Figure 4A) of 2-ME rats, in accord with gene expression, and no significant changes were observed in the frontal region (Figure 4B). In accord with what previously discussed about MAS1 role in hypotensive response, our data suggest that, in our model of SHRs undergoing two MEs, the involvement and augmentation of expression of Mas1 at frontal cortex level, might play an important role in the restoration of BP and cardiac parameters in SHRs. Differently, a significant augmentation of AT1R and ACE gene expression observed after two MEs in the parietal area (Figure 3A), might be attributed to a feedback response to the hypotensive effect induced by ME procedure, and possible reduction of AngII levels. Furthermore, in order to verify that observations in SHRs were effectively related to the hypertensive state, we performed an analogous investigation in a group of NT rats undergoing ME procedure. Interestingly, the gene expression analysis in NT rats revealed a very different pattern for the five studied genes both in parietal and frontal areas, when compared to SHRs. The complete non-detectability of AT1R in both areas of NT rats is in accord with its role as AngII principal receptor in pathological settings, mainly in hypertension. In parietal area, both AT2R and Mas1 were significantly increased in NT SO with respect to NT 2-ME, suggesting their important role in the preservation of normotensive conditions. Furthermore, in parietal area, ACE and ACE2 resulted N/D in NT SO rats, whereas there was some expression of these genes after 2-ME procedure (Figure 3B). Differently, in the frontal area of NT rats, no difference was observed in the expression of all genes tested between SO and 2-ME rats (Figure 3D). Therefore, (1) in the two brain areas studied RAS gene expression pattern is very different in SHRs with respect to NT rats and (2) 2-ME procedure supports RAS protective arm effects in chronic hypertensive conditions but not in NT subjects.

## Conclusion

Our data indicate that in our SHR model a double ME procedure induces a hypotensive response and that a modulation of gene expression and protein levels of main RAS components occur in cerebral cortex regions. In particular, in a translational perspective, a very interesting aspect of our study is that the hypotensive effects induced by the double ME persisted for the entire post-treatment observation period of 240 min. In future studies, it will be interesting to evaluate the effects of repetitions also in consecutive days in order to analyze how far the effects of repetitive ME will drive the hypotensive response. Moreover, to evaluate the effects of the same procedures after the treatment with specific inhibitors of ACE2 or MAS1 will add important information on involvement of the different RAS metabolites analyzed. In conclusion, on the basis of previous data on rats and humans and the present data on SHRs and NT rats, it is possible to speculate that possible new procedures, implying repetitive MEs, may find a clinical application as non-pharmacological ancillary aid in acute or chronic treatment of arterial hypertension.

## Author Contributions

LS and RS conceived the project. CC, DL, and GF performed the experiments. LS, CDS, SB, AC, GI, and RS contributed to discussions regarding the project. LS, CDS, and RS analyzed the data, wrote and revised the manuscript.

## Conflict of Interest Statement

The authors declare that the research was conducted in the absence of any commercial or financial relationships that could be construed as a potential conflict of interest.
